# Do local economic interests matter when regulating nationally significant infrastructure? The case of renewable energy infrastructure projects

**DOI:** 10.1177/0269094218763000

**Published:** 2018-03-19

**Authors:** Yvonne Rydin, Lucy Natarajan, Maria Lee, Simon Lock

**Affiliations:** University College London, UK

**Keywords:** evidence, infrastructure, local businesses, planning regulation, renewable energy

## Abstract

Government policy in the UK, as in many countries, sees investment in infrastructure projects – particularly large ones – as a key means of supporting the national economy. But where does this leave local economic interests in the loci of these projects? And how does the regulation of such projects handle these interests? These are the questions addressed by this paper in the context of renewable energy projects that are regulated by the Nationally Significant Infrastructure Projects regime. Drawing on original research into the regulation of 12 projects – and using thematic analysis of key documents and focus groups with local participants – the analysis highlights the limited understanding of the local economy presented, the challenges that local businesses face in participating and the partial protection offered to them. It concludes by proposing agendas for reforms and future research.

## Introduction

The promotion of big infrastructure projects has been a central plank of the UK central government’s economic policy, extending back to the pre-2005 New Labour governments and through the various governments since then. The first National Infrastructure Plan (HM Treasury and Infrastructure UK, 2010), though, was only published in 2010; described by Marshall (2013: 136) as ‘lightweight’, it was revised the following year. More recently, a National Infrastructure Delivery Plan (Infrastructure and Projects Authority, 2016) was published in 2016 covering the period to 2021 and underpinned by a National Infrastructure Pipeline to 2021. This covers projects worth over £480 billion, about 50% of which is accounted for by public investment. Outside of Government departments, a National Infrastructure Commission (NIC) was established as an executive agency of the Treasury in January 2017. Its role is to provide advice and make independent recommendations on infrastructure priorities. The goals of the NIC reveal the overarching goals of infrastructure policy:

- To support sustainable economic growth across all regions of the UK;
- To improve competitiveness; and
- To improve quality of life.

Given the emphasis on infrastructure investment supporting economic growth, there has been concern voiced that the planning system was inefficient in dealing with proposals for major infrastructure projects, causing unreasonable delays and excessive costs for all participants – developers, local authorities and central government (whose Planning Inspectors often chaired public local inquiries into the proposals). This has led to a new regime being established for regulating certain infrastructure projects in England and Wales, the Nationally Significant Infrastructure Projects (NSIPs) regime. This was established under the Planning Act 2008 (and modified by the Localism Act 2011 and the Infrastructure Act 2015 – see Marshall, 2013, for background).

Under this regime, all NSIPs (a category determined by set thresholds; in the case of energy projects these are generation capacities exceeding 50 MW for onshore and 100 MW for offshore projects^1^) are dealt with by a strictly time-limited regulatory process. This goes from consultation, environmental assessment and other pre-application work, through preparation for and involvement in an Examination under the direction of an Examining Authority (ExA, which may be an individual or a panel) appointed by the National Infrastructure division of the Planning Inspectorate, and then publication of an extensive report with recommendations from the ExA. The final decision is taken by the Secretary of State. The central stage of the Examination comprises a mixture of document exchange (representations, answers to questions from the ExA, drafted agreements and Development Consent Order, etc.) with public hearings and site visits. The whole process from acceptance of the application to decision must take no longer than 18 months and the Examination itself lasts six months at most.

Marshall and Cowell (2016) argue that the concern about the length of time that planning regulation of major infrastructure has taken in the past is not substantiated by historic data and they see the ‘reforms’, whose stated aim was to streamline and speed up the regulation of such projects, as an ideological response. They argue that the main impact has been to compress the period of public hearings, with potential implications for the ability of different interests to be heard. This marries with a further concern about the new regime that, even where a range of interests are able to make representations, the policy and legislative context may prevent them from carrying weight within the decision-making process. Decision-making on NSIPs is guided by central government policy, as set out in National Policy Statements (NPSs). These NPSs reiterate the need for new infrastructure from a national perspective and set out that development consent can only be refused on the basis of significant adverse impacts. It is notable that the vast majority of NIPS have been consented; only one renewable energy (RE) project – Navitus Bay Offshore Wind Farm – has been refused consent. In such a context, there has been concern expressed about the ability of voices critiquing the project, including from local interests, to have influence (Lee et al., 2012; Rydin et al., 2015).

There is, therefore, a tension between the pursuit of the national economic interest and giving local interests – including the local business interests that are the focus of this paper – a say in planning regulation of these major projects. It is this tension that sets the context for our research into major RE projects and their regulation. The Climate Change Act 2008 (HM Government, 2008) established statutory carbon reduction targets implying considerable investment in RE infrastructure. A Renewable Energy Strategy (Cm 7686) was published in 2009 (HM Government, 2009) and the UK Renewable Energy Roadmap (DECC, 2011) has been twice updated. While EU targets for the share of renewables within overall energy generation may have become less significant since the referendum vote in 2016 for the UK to leave the EU, the UK intends to continue to comply with international law on climate change, including the 2015 Paris Agreement. In any event, the investment pipeline for RE projects is substantial and is making its way through the NSIPs regulatory regime.

Our paper focuses on how local economic interests fare within the NSIPs regime where major RE projects are concerned. The NPSs recognise that any infrastructure project has to be sited in a particular location and will have local impacts, including those that are categorised as ‘socio-economic’. The empirical question we address is how these socio-economic impacts, particularly as they concerned local businesses, were handled at the regulatory stage within the NSIPs regime. This is a relatively neglected area of research. The majority of the work on planning systems and RE infrastructure has focussed on understanding the residents’ perspective on such schemes (Eltham et al., 2008; Firestone and Kempton, 2007; Firestone et al., 2012; Hall et al., 2013; Jones and Eiser, 2010; McLaren Loring, 2007) and the extent to which planning is responding to resistance to such infrastructure development from residential communities (Aitken et al., 2008; Anderson, 2013; Cowell, 2010; Gross, 2007; Van de Horst, 2007). Where planning studies look at the local business sector, it has tended to do so with regard to formal institutional structures (Fox-Rogers and Murphy, 2014), rather than their engagement with planning regulation. Our research therefore makes a distinctive contribution to understanding the planning regulation of RE infrastructure where local businesses are concerned.

The next section sets out the methodology of the research project. The analysis proceeds under two main headings: the local economic impacts of RE infrastructure projects and the engagement of the NSIPs process with local businesses and their concerns. The discussion is brought together in the conclusion.

## Methodology

The research presented here is drawn from a larger project on major RE infrastructure projects undertaken during 2015–17. This project considers local publics more generally and how their concerns and views are constructed as evidence within the NSIPs regime. It operates within a Science and Technology Studies (STS) conceptual framing that emphasises the constructed nature of evidence and the performative nature of the regulatory regime. The central insight of STS is that knowledge is generated and warranted through institutional processes and that key artefacts play a role in confirming what counts as knowledge (Latour, 1999). While originally focussed on scientific institutions, this approach can be applied in legal and regulatory contexts also (Latour, 2010; Van Oorschot and Schinkel, 2015). From an STS perspective the regulatory regime is seen as having performative dimensions in terms of creating the categories it discusses; in particular, the regime is implicated in deciding what counts as evidence sufficient to justify a decision. This is an important dimension of an evidence-based process such as the NSIPs regime and has implications for its outcomes. Here the artefact of the ExA report plays its part in establishing the rationalisation of decisions and recommendations on the basis of selected evidence presented during the Examination. For these reasons, study of ExA reports was a first stage in the methodology adopted.

This first stage focussed on 12 RE cases which had been through the NSIPs regime (see Table 1). For these, the ExA’s reports were read, coded (using 119 codes under the five headings of actors, impacts, evidence, policy and mitigation) and analysed. These reports are extensive documents amounting to several hundred pages each. The codes were selected following initial reading of a selection of reports and then added to as further reading revealed additional issues of interest; this is usual practice for thematic coding and analysis of texts. Coding within NVivo software then allowed repeated code runs alerting the researchers to points within the reports where local businesses and evidence on socio-economic impacts were discussed. Where reports are quoted from, the project and the page number are given; the full report can be accessed from the PINS website at: www.infrastructure.planninginspectorate.gov.uk/.

**Table 1.** RE infrastructure case studies and focus group details.

Case study	Scale of RE	Focus groups with local actors (those with businesses in bold)
Kentish Flats OWF Extension	10–17 turbines; 90–141 MW	N/A
Galloper OWF	Max 140 turbines; 504 MW	Residents: 5; Businesses: 0; Groups: 1
Burbo Bank OWF	Max 69 turbines; 259 MW	Residents: 2; Businesses: 0; Groups: 1
**Rampion OWF**	**Max 175 turbines; 700 MW**	**Residents: 2; Businesses: 1; Groups: 4**
**Walney OWF Extension**	**207 turbines; 750 MW**	**Residents: 1; Businesses: 2; Groups: 2**
Triton Knoll OWF	288 turbines; 1200 MW	N/A
**Navitus Bay OWF**	**Max 194 turbines; 970 MW (plus mitigation option)**	**Residents: 5; Businesses: 1; Groups: 7**
**Brechfa Forest West WF**	**Max 28 turbines; 84 MW**	**Residents: 1; Businesses: 1; Groups: 7**
Clocaenog Forest WF	Max 32 turbines; 96 MW	Residents: 5; Businesses: 0; Groups: 4
Swansea Bay Tidal Lagoon	240 MW	Residents: 1; Businesses: 0; Groups: 10
North Blyth Biomass Plant	99.9 MW	N/A
Rookery EFW Plant	65 MW	N/A

EFW: Energy from Waste; OWF: Offshore Wind Farm; WF: Wind Farm (onshore); RE: renewable energy.

In the second stage, for eight of these NSIPs (plus a London control case), focus groups were held to explore issues raised in more depth, with transcripts again coded and analysed. Local residents, businesses and groups who had participated in NSIP examinations (but not as an applicant) were identified from the Planning Inspectorate’s website and invited to take part in a focus group. Each event was held in the locality of the NSIP in question, during in the summer of 2016. People were asked about their experiences and views of NSIPs processes, and discussions were moderated around five themes: initially getting involved, giving evidence, engaging with others, their own influence and reflections on the experience overall. Given the focus of this paper, the analysis here draws on the contributions of local business representatives, who were present at four of our focus groups (see Table 1): three offshore wind farms (Navitus Bay, Rampion and Walney) and one onshore wind farm (Brechfa Forest). The confidentiality agreement with participants means that all quotes are anonymised but the nature of the business is indicated.

This research was supplemented by additional document analysis from the Planning Inspectorate website, selective attendance at ‘live’ cases and a range of national-level interviews with NGOs and relevant professionals. Two validation workshops were also held towards the end of the project to discuss key findings and recommendations with a selection of ExAs and a group of infrastructure developers. In the analysis below, the discussion of local economic impacts draws largely on the analysis of ExA reports, set in the context of other relevant literature; the longer discussion of the engagement of the NSIPs process with local businesses and their concerns also draws on the focus group discussions.

## The local economic impacts of RE infrastructure projects

The entire rationale of the government’s infrastructure policy and the establishment of the NSIPs regime is that such infrastructure generates important economic benefits and is essential for national economic growth. But there is also often an assumption that these projects will produce local economic benefits. As one of the business representatives in our focus groups stated: ‘It’s difficult though because there is a lot of an economic benefit from this kind of things in the local area’ (Ferry company, Walney OWF).

Yet these benefits can be overstated. The anticipated numbers of construction jobs cited by applicants in our case studies ranged from 35 to 1850 (although the latter included works outside the project site) and these jobs would only be for the period of construction; longer term operation and maintenance (O&M) jobs were far fewer, with 4–250 expected jobs reported (see Table 2).

**Table 2.** Direct employment from RE case studies (where given).

Case	Project construction jobs	Operation & Maintenance (O&M) jobs
Galloper OWF	850	
Burbo Bank OWF	1545 person yrs	155 person yrs
Rampion OWF	500	65–85
Walney OWF Extension	230	185
Navitus Bay OWF	35–890 across three scenarios	10–250 across three scenarios
Brechfa Forest West WF	150	4–5
Swansea Bay Tidal Lagoon	1850 (incl. offsite works)	81
North Blyth Biomass Plant	Peaks at 300; average of 150	50–60
Rookery South EFW Plant	320	80

EFW: Energy from Waste; OWF: Offshore Wind Farm; WF: Wind Farm (onshore); RE: renewable energy.

There is also the issue of whether these jobs would be filled by local people. While generalised claims of local employment possibilities were made, it was often recognised that the skills needed might require workers from outside the locality. In case of the Rookery South EFW plant, while it was argued by the applicant that all jobs were likely to be suitable for locals, they admitted that some specialist skills would be required from outside the local labour pool; for the extension to the Walney OWF, it was baldly stated by the applicant that these jobs were not likely to be suitable for locals.

Rather than focus on the limited jobs directly created by the projects, applicants typically argued that there would be a positive impact on the local economy through the supply chain. Sometimes this amounted to a rather generalised reference to multiplier effects (e.g. Rookery South EFW Plant); but, in other cases, more specific economic linkages were claimed and discussed. For the North Blyth Biomass Plant, a commitment was made to develop a register of local firms for construction work and in the case of Clocaenog WF, the applicant set out measures that would be deployed to encourage the use of local suppliers and local recruitment. The impact of these measures was not, however, assured and, in the latter case, the ExA concluded with the rather weak claim: ‘Whilst it cannot be guaranteed that all future employment on the project would benefit local people, these measures would ensure that opportunities are fully publicised’ (ExA report, p. 91).

This fits with the research literature looking at the employment effects of RE projects; this suggests that such projects do not, in themselves, necessarily create significant employment in the locality of their siting or ensure that these jobs would be filled by local people (Callaghan and Williams, 2014; Okkonen and Lehtonen, 2016; Whyman, 2015). Much of this work has been done in the USA context; for example, an integrated assessment for offshore wind energy in Lake Michigan saw ‘little agreement on the benefits to coastal communities’ (Nordman et al., 2014: 287) while a comparative study of shale gas and wind generation in Texas did not find a statistically significant local impact from the wind industry (Hartley et al., 2015). Another Texan study makes it clear that ‘that the level of impacts and their distribution [from wind energy] depends a great deal on the extent to which the locality of interest is able to directly participation in the construction, operation and ownership of a project’ (Slattery et al., 2011: 7939), highlighting the importance of a local supply chain. These seem to be largely missing in the English and Welsh contexts.

In the absence of evidence of such supply linkages, often there is reliance within the NSIPs regulatory process on memoranda and agreements signalling the goodwill of the applicant towards local employment and procurement. In the case of the North Blyth Biomass Plant, the preparation of a Workforce Development Strategy was secured by a condition in Development Consent Order as the ExA thought this ‘should maximise the opportunities for local employment benefit’ (ExA Report, p. 32); for the extension to the Walney OWF, key parties agreed to prepare a Memorandum of Understanding on Economic Cooperation to cover training and supply chain issues. However, such memoranda have limited force.

In two of our cases, the ExAs were not entirely happy with the efforts made by the applicant to secure local employment and supply chain benefits. The Rampion OWF case referenced a supply chain project led by Marine South East seeking to ensure local employment and enhance local skills for broader local economic regeneration. However, the ExA was concerned that this was not secured within the Development Consent Order, formally linking the planning permission to the supply chain project. Eventually the local authority, West Sussex County Council accepted the lack of a formal link and the ExA did not pursue the matter further, stating that it was ‘convinced’ that the applicant was working with local authorities on this issue. In the Galloper OWF, there was considerable discussion led by the ExA about the lack of specific measures for maximising opportunities for local business, local employment and training. However, their attempts to explore these issues did not get much response. It was simply stated that, because of the characteristics of the industry’s supply chain, a relatively small percentage would be based in Suffolk or even the East of England region (ExA Report, p. 174). The end result was again an Economic Memorandum of Understanding signed by the applicant and the local authority and reliance on an existing Skills-for-Energy initiative run by local government to promote relevant training.

In the case of OWFs, part of the problem in linking projects to local employment and procurement opportunities lies in the fact that the location of the ports to be used for construction activity and for O&M is not identified at the time of the regulatory decision-making, often to maintain the competitive position of the wind developer. This severely limits the specificity of the socio-economic impacts analysis. In the case of Galloper OWF, even when the ExA Panel pressed, no commitment was made on the shore-base for the project and it was acknowledged that the socio-economic impacts could only be assessed ‘at a high level’. When considering the Rampion OWF, the ExA said that the discussion about the location of the construction port ‘did not provide clarity’; while they considered that the socio-economic assessment was adequate despite this, they did go on to say that the economic impacts could not be given much weight if there was no commitment to a specific location for the port. Again, in the case of Burbo Bank OWF, because the ports for construction, O&M activities had not been identified, the spatial distribution of any economic benefits was not certain and these could not, therefore, be taken into account.

Often the economic analysis moves upscale. When examining the Walney OWF Extension, emphasis was laid on the positive employment impact at the regional and national scales, given that it was not possible to determine the local impact. This echoes the findings of Kerr et al. (2014). They noted the lack of evidence on job creation and supply chain effects of new infrastructure projects and that existing research was only available at regional scales and was ‘too general’ (p. 696). Local-scale datasets were absent, including the existing geographic distribution of jobs, and the displacement of jobs was particularly poorly understood.

So, if the direct positive economic potential of these NSIPs within the locality of the projects may be small, difficult to assess, little researched and often given little weight in deliberations, what of other potential socio-economic impacts? In particular, how are existing local businesses affected? As Okkonen and Lehtonen (2016: 826) state: ‘In renewable energy projects, there is often a problem at the level of local communities because they are left with negative externalities while most of the revenues and benefits leak to non-local actors.’ There are three main sectors potentially impacted by these RE projects: tourism (which is affected by on-land and offshore projects and includes for example hotel and catering businesses, as well as leisure provision such as horse riding and diving) and fisheries and ferry services (impacted by offshore wind farms and other marine renewables projects).

Local tourist businesses are concerned that these major projects will deter visitors because of the visual impact or noise generated. There is a prevailing view that tourism depends on unspoilt landscapes and that RE infrastructure can introduce elements of an industrial scene (Rudolph, 2014). A study of tourism impacts of wind farms in Scotland suggested that, contrary to earlier research reviews, ‘the impact is sufficiently large to suggest specific action may be required’ (Riddington et al., 2010: 250). In addition, there are effects such as safety issues in the vicinity of turbines affecting the ability to get insurance for tourism businesses (reported for horse-riding businesses in the Brechfa WF focus group). The impact on local fishing businesses arises from the disruption during the construction period; but, once in place, offshore RE projects can continue to affect fishing activities. Kermagoret et al. (2014) summarise the impacts as loss of fishing area and access, changes in water quality during construction affecting the benthic ecology, further ecological impacts during operation and navigational hazards arising from cabling. Finally, in some locations, ferry services run across potential offshore wind farm locations; as with fishing vessels, this can affect the routes that the ferries can take with consequent economic impact.

Given this pattern of uncertain positive local economic impacts and potential negative impacts on specific local businesses, how such issues are considered and responded to within the NSIPs regulatory process?

## The engagement of the NSIPs process with local businesses and their concerns

Drawing on the focus group data as well as the ExA reports, this section considers the NSIPs regime in terms of direct local business involvement, handling of evidence on local economic impacts, outcomes concerning local businesses and some outstanding problematic issues.

### Local businesses’ involvement in the NSIPs process

The involvement of local business representatives in the regulatory process can be considered in terms of pre-application consultation, engagement with the documentation provided and attendance at the hearings themselves.

It is a requirement of the NSIPs regime that the developer undertakes consultation with local stakeholders and submits a statement on that consultation as part of the application process. Thus, there is typically a degree of engagement with local businesses as part of this. However, not all local business interests are equally engaged. In the case of offshore wind, there has been an emphasis on targeting local fishing interests. Representatives of local fishermen in the focus groups detailed how they were actively sought out:

We were approached directly by a mediating company to be a mediator between the local fishermen to attend a fishing meeting. … We probably have somewhere between 3 and 6 emails a week. (Fishing business, Rampion OWF)

We do get a lot of consultation. We get lots of information. Week after week people gives us lots of things. (Fishing business, Walney OWF)

But they also noted during the focus group discussion, that this was not a universal experience: ‘I stupidly assumed that everybody around the table would have the same level of communication as we still currently do’ (Fishing business, Rampion OWF).

There was a general feeling that this was largely a ‘tick box’ exercise: ‘And sometimes you do get the feeling that all this consultation is just to tick a box to say they’ve gone through consultation’ (Ferry company, Walney OWF).

This related to concerns that the consultation was not being carried out in a way that was most meaningful to the local interests. The reliance on email and scheduled meetings was an example of this:

When DONG Energy first fell foul, it was simply because lots of fishermen are not great in filling forms and paper work. And they were asking for meetings. And the fishermen wouldn’t attend … Fishermen are not good in IT. They just want to fish. (Fishing business, Walney OWF)

But the people I have represented for this, I can assure you, some of them don’t have email addresses. A lot of them … if it wasn’t for someone representing them … a lot would never have known about this, wouldn’t never had any communication with E ON or mediators at all, the planning at all … and they would have their livelihoods turned upside down. … (Fishing business, Rampion OWF)

The use of a local fisherman to act as a mediator was one way of overcoming these problems:

… the fishermen had this representative XXXX who, he was paid by DONG Energy. … DONG Energy were clever, because they knew that the fishermen, who could not even write 1 or 2, could talk to XXXX, cos they knew he was fisherman. They were very clever employing XXXX. (Fishing business, Walney OWF)

This was not a universal approach though.

The concerns about the means of communication became even more pronounced when the scale of documentation associated with an NSIP project was discussed:

What I was going to say is about the process, the documentation… The sheer volume was biased towards the big organisation who got a huge team behind them. The likes of Challenge Navitus [a local NGO] were amazing. But an individual, I mean, I don’t have … no hope at all … (Boat charter business, Navitus Bay OWF)

… it is not they ignore you; in fact they are very good in not ignoring you, you know. But sometimes that’s the issue because they bombard you with so much stuff that [it] is almost impossible to keep up. (Ferry company, Walney OWF)

Furthermore, much of this documentation is distributed and made available by electronic means, echoing the reliance on emails.

And there was a pervading sense of imbalance in terms of the production of this documentation:

And if you spend all day writing the best email you’ve written to them, rest assure they have at least 15 people sat in an office, who will be far better [on] the subject, who will be far better equipped. They have far more money to send you a much better reply to you. (Ferry company, Walney OWF)

The biggest imbalances though occurred in the hearings, where the norms of public speaking put local businesses under considerable psychological pressure. This was put eloquently in the case of the Navitus Bay OWF proposal:

… diving, I don’t think it was going to come up at all, although it was programmed to. And I had arranged to meet my wife at 6 o’ clock. And at 5.30 pm I said I’ll meet you at 6. Ten to six, diving came up [laughter]. And I was in an absolute panic. If anybody remembers it, because I stood up with the microphone and I … this is like the first time ever … and I had to explain my point. I had a mental block after about 30 seconds which was horrible. (Boat charter business, Navitus Bay OWF)

In this case, the ExA encouraged him and: ‘I was able then to get on and I walked out about 6 o ‘clock [laughter] having said what I needed to. But I did find it incredibly daunting’ (Boat charter business, Navitus Bay OWF).

Even when local business people, despite these challenges, felt that they had had their say in these hearings, there was the question of whether they had been heard: ‘I do feel that we had an opportunity to say everything that we wanted to say but I don’t necessarily feel like it was heard all the time’ (Fishing business, Rampion PWF).

Some local business interests considered employing a representative in response to the imbalances of the process as they experienced it:

I think we wondered if whether we should actually employ someone full-time just to fight our corner. And in the end we didn’t … We stepped back. It’s just so time-consuming to really fight it as well as you should. Especially when it is not your full time job. It’s their full time job but it is none of us full time job. You always feel a bit disadvantaged somehow. (Ferry company, Walney OWF)

Thus, the engagement through consultation, document exchange and attendance at the hearings was seen as problematic from the local businesses’ perspective, making assumptions of time, e-literacy and public speaking skills that local business representatives often did not have.

### Evidence concerning impacts on local businesses

But representations per se do not necessarily have an impact on the discussions and decision-making within the NSIPs regime. The conclusions of the ExA have to be evidence based and yet there is a relative lack of evidence offered on such impacts compared, say, with landscape/seascape or ecology concerns. The evidence offered is also often considered to be lacking in robustness (Pieraccini and Cardwell, 2016) and this applies to evidence offered both by the applicant and by local businesses. In the Rampion OWF case, ‘the Panel was struck by the very limited evidence regarding socio-econ matters presented’ (ExA Report, p. 144) and the local authority proved unable to comment when questions were raised on certain matters due to ‘a lack of robust evidence’. Speculative suggestions, such as that this wind farm could become a tourist attraction if a visitor centre provided, were largely discounted because of the absence of any evidence on or certainty about the positive impact (ExA Report, p. 148).

In most cases, concerns were expressed in the Examination that the NSIP project would detrimentally affect local tourism.^2^ But it was difficult to produce compelling evidence about the relationship between the NSIP project and tourism revenues or employment. In the Clocaenog WF case, the shortage of material on this causal relationship between wind farms and tourism was specifically noted. The applicant provided a literature review which only pointed to the absence of any ‘evidence to suggest a serious negative impact’ (ExA Report, p. 90). The Brechfa Forest West WF also raised concerns about the cumulative impact of multiple wind farms on local tourism but, despite the offer of survey-based evidence by the Carmarthenshire Tourism Association, the detail on this sector was deemed to be insufficient; the ExA commented: ‘I consider none of the evidence to be compelling’ (ExA Report, p. 92).

The Navitus Bay case saw discussion about the use of visitor perception studies as a means of predicting the economic impact of the OWF. These studies suggested that visitors came to the area because of the coast and sea views and a percentage of those surveyed said they were less likely to visit if the OWF was developed. The ExA Report suggested that part of this might be translated into action and that, therefore, these surveys could be given some weight. However, it remained difficult to link cause and effect where the impact of wind farms on tourism was concerned and, in particular, it was not possible to quantify this effect. This contrasts with other issues, such as ecological impacts, where quantification dominates the discussion of impacts (Rydin et al., in press).

The focus groups also revealed concerns that evidence on tourism was being manipulated; while it is not possible to substantiate (or refute) these claims, this is a notable feature affecting the trust of local business interests in the NSIPs regime. Talking about a project proposed for the Clocaenog Mountain, a local business representative said:

… the person who did the Tourism Assessment designated a crossroads on the mountains … and then used software to work out the route by road from that crossroad to all of the properties. So you had properties that they were going to suffer from shadow flicker were being described as being 3 and 4 km away from the wind farm site. Because she manipulated data to that extent. And that really shouldn’t be allowed. (Tourism business, Brechfa WF)

And on the Navitus Bay OWF case:

So, I actually found information in there that was totally wrong. They supposedly did a survey and it’s in black & white. In details it says ‘3000 divers a year’ and basically the impact was ‘insignificant’ … I went to the pier. I spoke to XXXX who is the Treasurer and I said ‘XXXX, do you keep records?’ And he said ‘Yes’ and I explained what I wanted and about one day he came back and he had actual receipts for the previous year of 10,854, a figure similar to that. I thought, well this is totally ludicrous of what they were saying. When I did challenge Navitus Bay, they very quietly said, I think the figure is 30000. … They dropped a nought off. So that was the level of the information they were publishing, which is not good. (Boat charter business, Navitus Bay OWF)

Similar concerns were expressed regarding the inconsistency with which socio-economic evidence seemed to be treated:

I found it interesting, if you say compare Brechfa West against Llanllwni Mountain, that two different Inspectors, given the same piece of evidence accepted it as granted in one case and refused it on the other; or, depending on what outcome they wanted. I got the impression, whether they wanted to refuse planning commission those arguments were accepted; where they wanted to grant planning permission those arguments were refused. But it was the same argument in both case. (Tourism business, Brechfa WF)

The absence of evidence or doubts about the evidence result in little weight being placed on these impacts on the local economy, as revealed by the ExA’s reports. In the case of the Kentish Flats OWF Extension, the Local Impact Report was considered, in parts, to be ‘speculative and over-optimistic’ (ExA Report, p. 120) and thus not to be relied on. In Brechfa Forest West WF, the uncertainties around tourism impact compounded the problems of limited information on the effects of the construction and O&M activities on local employment, downplaying the weight given to socio-economic impacts. For Burbo Bank OWF, the socio-economic effects were described as ‘uncertain but likely to be neutral’ with ‘no conclusive evidence’ of adverse effect (ExA Report, pp. 88–89).

In effect, attention within the regulatory process turns elsewhere because the evidence is constructed as insufficient and insufficiently robust. In documentary terms, this is shown by the limited number of pages devoted to the local economy within reports that typically extend to several hundred pages; on average, in our sample, the proportion of pages devoted to socio-economic impacts (including those specifically on commercial fishing interests) was 5% (ranging between 2 and 10%). While it is repeatedly stated that there is ‘limited weight to be given to assertions of socio-economic impacts that are not supported by evidence’ (Burbo Bank ExA Report, p. 89), there is little attempt to force the production of knowledge to resolve these uncertainties. Indeed the Triton Knoll OWF case saw it stated that: ‘legally such information only has to be provided in relation to likely significant effects based on an assessment carried out within the bounds of current knowledge’ (ExA Report, p. 96). So impacts on the local economy are uncertain and lacking an evidence base but this is accepted as the state of affairs, resulting in limited discussion of these impacts and a focus of regulatory attention elsewhere.

This sparse discussion of socio-economic impacts can be contrasted with the much more detailed discussion of ecological impacts. The contrast is especially striking when comparing marine species and fish populations as an ecological and as a socio-economic issue. The structural reason for the more detailed discussion is that EU Directives require decision makers to make a substantive judgement on ecological impact, rather than just to take the issue into account. Thus, there is considerable discussion in the ExA Reports about how far certain fish populations will be affected by construction and O&M. But that is not translated into a lengthy consideration of the economic impact of changing fish populations on local fishing industries. From the local fisheries’ perspective, the lack of strong evidence on local business impacts was contrasted with the time and effort taken to quantify impacts on ecology. Fish stocks were seen as of more interest than fish catches, not to mention birds:

Then, I started looking into a bit more detail and I found that NBDL [the developer] had done an enormous amount of work for everything regarding our fish, crustaceans, cetaceans, whatever you like. Nothing about humans. There was absolutely zero about humans. (Boat charter business, Navitus Bay OWF)

… it always seems to be the bird people who get the absolute top priority. (Ferry company, Walney OWF)

### Outcomes of the NSIPs process concerning local businesses

When considering the outcomes of the NSIPs process, the decision to consent or not is of less interest than the detail of the decision since, given the strong policy steer on the need for such infrastructure, refusal of consent is an unlikely outcome. This was repeatedly recognised by local business interests in the focus groups: ‘I think, to be honest, you just get a feeling generally, that these things are going to happen, and it is just about trying to get the least worst result out of it’ (Ferry company, Walney OWF).

Therefore, the focus shifts to changes in the proposed development which could mitigate impacts. For business interests, with their predominantly economic rationale, financial compensation can be an important means of mitigation. This can take the form of general community funds but, particularly for local fishing interests, there can be targeted compensation. As was noted in a focus group: ‘They were keen to pay compensation because … if we continued doing what we were doing, they couldn’t carry on doing what they were doing’ (Fishing business, Walney OWF).

And there was an acceptance that compensation might be the second-best outcome:

‘The money … it comes to a point where you decide, ok, you are not going to win here. What’s the best you can get out of it?’ (Ferry company, Walney OWF).

The recognition of the economic impact of temporary disruption to fishing activities during construction, and potential longer term effects on fishing, has led to established arrangements for compensation agreements. These are typically secured by Statements of Common Ground between fishery representatives and the applicant during the Examination of the project. In the case of the Rampion OWF, disruption payments were offered to cover loss of income and, in the Kentish Flats OWF Extension, funding for diversification of business activity was offered. But although routine, compensation is not automatic. Interestingly, in the Galloper case, the applicant resisted demands for compensation on the basis that there was no legal right to such compensation. These UK arrangements for compensation can be contrasted with that operating in France (Kermagoret et al., 2014) where an annual tax on offshore wind farms generates a fund that is redistributed to the local area, including directly to local fishing businesses (but not local tourist businesses); in addition, there are more specific compensation measures for particular impacts, providing these can be evidenced.

Alongside compensation agreements, there are often proposals agreed during the Examination for governing the ongoing coexistence of the project and local businesses, secured by agreements between parties. Again, this is particularly relevant to local fisheries. Governance protocols have been developed at the national level for engaging with local fishing interests, through the Fisheries Liaison with Offshore Wind and Wet Renewables (FLOWW) group which meets regularly in order to advance the relationship between RE developers and the local fishing industries; this has developed guidance on how this relationship should be fostered in the location of specific projects, during not just the regulatory process but also the operational lifetime of the infrastructure (De Groot et al., 2014). As a result, a typical set of governance arrangements that is put in place locally includes the appointment of a Fisheries Liaison Officer, establishing a Co-existence Strategy and devising a Fisheries Liaison Plan. There is also some emphasis on building trust between the applicant and the local fisheries, given the need for the project and the local businesses to work alongside each other. In the Burbo Bank case it was approvingly noted by the ExA that the applicant had ‘taken positive steps to foster good relationships and to make cooperation agreements’ (para 4.194, p. 85).

Often, the ExAs seem to assume that all the provisions for proper management, operation and construction, designed to reduce the risk to fishermen, will indeed be carried out by the applicant; in effect, they trust the applicant. Yet monitoring of the implementation of these provisions can be resisted by the applicant as too expensive, difficult and unreliable. This raises interesting questions about the reliability of enforcement and other means of ensuring that this trust is justified. There is a contrast here again with practice in France, where protocols for monitoring fish stocks have been negotiated with the fishing industry (Kermagoret et al., 2014: 200).

### Outstanding problems within the regulatory process

The recourse to agreements on compensation and future governance arrangements was not seen, within our focus groups, as sufficient to deal with the problems that such infrastructure posed to local business interests, particularly over the longer term. Two aspects of this longer term perspective were identified: the ‘creep effect’ of multiple projects; and the way that the local economy was being restructured by these projects.

The ‘creep effect’ refers to a common problem in planning regulation whereby decisions are taken project by project and the aggregate impact of several projects is often under-considered. The Environmental Assessment process is intended to consider aggregate effects but this often proves very difficult to do; the problems of modelling impacts on a project-by-project basis are multiplied when the combined effect of different projects is considered. This was very clear to local business interests: ‘And it’s getting bigger and bigger. And the fishing grounds are getting smaller and smaller. And as it’s getting bigger and … we found our voices is getting less and less’ (Fishing business, Walney OWF).

The regulatory process, operating on a project-by-project basis – even with some attempt at considering cumulative and aggregate impacts – finds it very difficult to deal with these issues to the satisfaction of local business interests. Furthermore, they see this ‘creep’ of infrastructure investment as linked to a restructuring of the local economy that these projects precipitate. Local businesses then become concerned that they do not have an economic future locally.

The threat can come from the marginal shifts in the local economy that have a significant impact on specific businesses. The fragility of local businesses in the face of the economic changes associated with such infrastructure projects (Rudolph, 2014) is sometimes acknowledged in the ExA’s reports. This was explicitly discussed in Rampion case (ExA Report, p. 151) and, at more length, in the Navitus Bay case; in the latter the ExA disputed the applicant’s claim that a volatile sector, such as tourism, must be robust. They found that tourist employment was sensitive to reductions in turnover and that numbers of business failures may not be an adequate indicator of the local economic impact (ExA Report, pp. 279–281).

However, infrastructure projects may also open up alternative commercial opportunities for local businesses. For the ferry interests in the Walney case, the shift in business opportunities was seen as a lucrative side effect:

Part of our business, we run ferries ourselves, back and forth, and part of it we also rent them out to other people. So we rented this ship out to XXXX for a year and a bit.

Actually this ship, it went from doing nothing to a charter rate. It was probably at the time, two and a half times what we could have expected to get in the normal chartering world. And after the end of the project, with a very drunken … from XXXX who proceeded to tell me that if our price from the beginning was twice as that, it could have still worked. (Ferry company, Walney OWF)

But for local fisheries, new commercial opportunities could result in them abandoning their original activities:

I know certainly other fishing towns and coast communities, now basically fishing is not existent with wind offshore farms. Because all the decent fishermen, decent skippers, have turned into skippers for the windfarms. They are not fishing any more.

But the fishing soul and the original community that was there, it is not going to be there any longer. (Fishing business, Rampion OWF)

This might be considered a positive economic outcome from the RE infrastructure investment, but local businesses see it as driving change in the local area more generally, change which is not necessarily beneficial and is certainly poorly understood and planned for.

## Conclusion: Local businesses and RE NSIPs

We have addressed the question of how local economic interests fare within the NSIPs regulatory regime where RE projects are concerned. The regulatory process for these projects operates in the context of a very strong policy steer from central government in favour of consent, on the basis that this contributes to national economic growth. This puts local economic interests in a weak position within that regulatory process. We have seen, though, how the detailed implementation of the regulation creates further problems for local businesses in the vicinity of proposed NSIPs.

The direct beneficial economic effects of the projects in the locality are typically low and often uncertain. Applicants make very vague commitments concerning local employment and supply chains. This is exacerbated by analysis of socio-economic impacts at too high a level spatially (at the subregion, region or even nation), lacking the granularity required for understanding local impacts. The fact that the O&M ports for OWFs are not specified further adds to the lack of knowledge about likely impacts.

Consultation with local business interest is uneven; it seems that fishing interests fare better than other local business sectors such as tourism. The impact of national-level attention to such consultation through the FLOWW process can be readily seen. However, all businesses reported concerns about the consultation process. There was an over-reliance on email, online documentation (and too much of it) and formal meetings, none of which mesh well with local business’s everyday lives. It was notable that employing a local fisherman to support the consultation was considered ‘clever’ by other local fishermen.

When it came to the Examination itself, local business representatives often found speaking at a hearing very daunting, notwithstanding a supportive attitude from ExAs. The evidence that was presented on socio-economic impacts was often absent, of minimal quality and quantity and judged not to be robust. The lack of quantification was an issue in the context of considerable quantified evidence on, say, ecological impacts. There were perceived concerns about data manipulation and lack of consistency in how socio-economic impact evidence was treated; while these concerns cannot be verified, they point to a lack of trust in the regulatory process.

Discussion of socio-economic impacts does lead to mitigation measures, notably compensation offers. However, these were focussed more on the fishing industries, again reflecting the impact of the FLOWW process. There was frequent recourse to promises of governance measures for coexistence of the infrastructure and local businesses, but it is not yet clear how these will work and how effective they will be. Local businesses expressed concern that the cumulative effects of multiple infrastructure projects on local businesses and the restructuring of the local economy more generally were not being adequately considered.

This research points to the structural power imbalance within the regulatory context between the local economic interests in the vicinity of RE infrastructure development and the infrastructure industry itself. Focussing just on the fishing industry – which we have seen fares better than other local business sectors – Gray et al. (2005) point to the fundamental power imbalance between a highly fragmented fishing industry (and the same point may be made about tourism and ferry industries) and the ‘superior strength’ (p. 138) of the RE industry. Aitken et al. (2008) makes a similar point with regard to onshore wind and the imbalance between developers and local interests more generally.

If one is to consider potential ways to enhance the power of local businesses, this research suggests the following would be fruitful areas to explore. The first three relate to how local businesses are engaged in the regulatory process; the last three relate to the evidence base on socio-economic impacts.

1. Establish protocols for involving local businesses beyond the fishing industry.
2. Consider employing local business representatives to act as liaison points.
3. Consider training and other support to local business representatives for attendance at hearings.
4. Invest in research on the socio-economic impacts of RE infrastructure, including better data and improved assessment methodologies at the local level, to support Local Impact REports.
5. Develop better methodologies for assessing the cumulative socio-economic impacts of incrementally expanding RE infrastructure.
6. Explicitly consider the longer term cumulative impact of RE infrastructure on the local economy and community.

Turning to the academic research agenda, this project has shown the value of exploring the role of local business interests within the planning process, particularly where these local business interests are typically SMEs rather than the major economic actors that planning studies so often focus on, contrasting their power with that of local residential communities. Regulatory processes can be a particularly revealing focus point for such research and it would be interesting to complement this study of the NSIPs process with an equivalent one of local planning regimes. Focus groups have proved an insightful methodology for exploring local business concerns, but given the small numbers always involved in such research, replication with further focus group research would be welcome. Such work would give local businesses a voice which, in the case of major infrastructure projects, is often muted.

## Notes

1 In 2016 all onshore wind projects in England were returned to the local planning system.

2 The Swansea Bay Lagoon was an atypical case in that the infrastructure investment was argued to actively open up sea views and offer water sports opportunities, thus contributing to tourism revenue and, for this reason, there was not universal but widespread support for this project locally.
